# Exploring MAPK and mTOR Pathways in Feline Thyroid Tumors

**DOI:** 10.3390/vetsci12070617

**Published:** 2025-06-24

**Authors:** Alexandra Monteiro, Tiago Bordeira Gaspar, Inês Borges, Sule Canberk, Mafalda Pinto, Isabel Pires, Paula Soares, Catarina Tavares

**Affiliations:** 1 Cancer Signalling & Metabolism, i3S—Instituto de Investigação e Inovação em Saúde, 4200-135 Porto, Portugal; axmonteiro96@gmail.com (A.M.); tgaspar@ipatimup.pt (T.B.G.); scanberk@ipatimup.pt (S.C.); mafaldap@ipatimup.pt (M.P.); psoares@ipatimup.pt (P.S.); 2 Cancer Signalling & Metabolism, IPATIMUP—Instituto de Patologia e Imunologia Molecular da Universidade do Porto, 4200-135 Porto, Portugal; 3 Department of Biology and Environment, UTAD—University of Trás-os-Montes e Alto Douro, 5000-801 Vila Real, Portugal; 4 CIVG—Vasco da Gama Research Centre, Department of Veterinary Sciences, Vasco da Gama University School, 3020-210 Coimbra, Portugal; 5 CEDIVET—Laboratório Clínico Veterinário, Leça do Balio, 4465-671 Matosinhos, Portugal; ines.borges@cedivet.pt; 6 Department of Veterinary Sciences, UTAD—University of Trás-os-Montes e Alto Douro, 5000-801 Vila Real, Portugal; ipires@utad.pt; 7 CECAV—Animal and Veterinary Research Centre, UTAD—University of Trás-os-Montes e Alto Douro, 5000-801 Vila Real, Portugal; 8 Department of Pathology, FMUP—Faculty of Medicine of the University of Porto, 4200-319 Porto, Portugal

**Keywords:** cats, immunohistochemistry, MAPK, mTOR, PCR, thyroid neoplasms

## Abstract

Thyroid tumors are common in humans and cats, occurring most commonly as benign lesions, whereas thyroid carcinoma is barely detected in both species. Determining the mutational status of MAPK-related genes (*BRAF*, *NRAS*, *HRAS*, and *KRAS*) and the activation status of MAPK and mTOR pathways is crucial for establishing the diagnosis, treatment, and prognosis of human patients. So far, the role of such players in feline thyroid tumorigenesis remains underexplored. This study aims to elucidate the presence and implications of potential shared molecular mechanisms between human and feline thyroid tumors. Fifteen formalin-fixed paraffin-embedded feline thyroid epithelial tumors (four tumors with atypia and 11 with no atypia) were collected to perform mutational and immunohistochemical analyses. Sanger sequencing targeting human homologous hotspots revealed no mutations in *BRAF* (human codon 600) or *RAS* (human codon 61) regions. A *KRAS* missense mutation (p.Gln232His) was identified in two tumors with no atypia of follicular pattern (2/15, 13%). Regardless of the mutational status, pERK (Thr202/Ty204) was immuno-expressed in 10/11 (91%), pS6 (Ser235/236) in 100%, and pAKT (Ser473) in 8/11 (73%) of the tumors with no atypia. The expression patterns of pERK, pS6, and pAKT and their associations with clinical-pathological features seem to mirror the progression dynamics observed in human thyroid tumorigenesis. pAKT expression was associated with the presence of multiple tumor foci within the same thyroid lobe, suggesting its potential as a marker of aggressiveness in feline thyroid tumors. This study introduces cats as potential animal models for human thyroid tumorigenesis, with further research required to confirm such potential.

## Simple Summary

Thyroid tumors commonly occur as benign growths in both humans and cats, with malignant cases being rare. This study analyzed feline thyroid tumors to identify molecular features equivalent to those found in human tumors, focusing on key gene mutations and protein activities involved in tumor development. Although common human mutations were absent, a rare mutation was detected in some feline tumors. Protein activity patterns in cats closely resembled those seen in humans, especially in more atypical tumors. One protein’s presence was linked to multiple tumor sites in the same thyroid lobe, suggesting it may indicate tumor aggressiveness. These findings highlight the potential of cats as comparative models for human thyroid tumors, which could improve diagnosis and treatment for both species, pending further research.

## 1. Introduction

Thyroid carcinoma (TC) is a rare form of thyroid disease in cats. In contrast, benign tumors are frequently found in hyperthyroid cats, and these account for 10% of adult feline tumors [1,2]. Adenomas are the most prevalent thyroid neoplasia in both cats and humans, accounting for up to 98% and 90% of cases, respectively [1,3,4,5,6,7]. Follicular adenomas (FA) emerge as the most common histotype of both species [1,4].

While molecular studies on feline thyroid tumors are lacking, research on feline hyperthyroidism has identified *TSHR* and *GNAS* mutations, leading to the proposal of cats as a spontaneous animal model for the human toxic nodular goiter [8,9,10]. In both conditions, thyroid nodule enlargement results from chronic activation of the adenylate cyclase–cAMP cascade, driven by these mutations, ultimately leading to excessive autonomous thyroid hormone production [8,9,10]. The value of cats as an animal model of human diseases has also been extended to oncology, concerning feline mammary and oral squamous cell carcinomas [11,12]. Still, the potential of cats as animal models in the context of human thyroid tumors has never been explored, and discoveries made in such matters might be important to improve the outcome of both feline and human patients. Comparative studies may pave the way for targeted therapies and personalized treatment approaches, thereby improving clinical outcomes, particularly for feline patients, which have limited treatment options in veterinary medicine. Currently, however, surgical excision and radioiodine therapy stand as the only options available in veterinary medicine. The latter is expensive and less available, and surgical excision might be challenging for the cases that present larger tumors or multiple tumor foci [13,14,15].

The mechanisms underlying tumor formation and development in cats’ thyroids are underexplored. In contrast, in human medicine, the molecular characterization of thyroid tumor-ascertained mutations in *BRAF*, *NRAS*, *HRAS*, and *KRAS* is relevant as they are players implicated in thyroid tumor progression through their activation of MAPK- and mTOR-signaling pathways [16,17,18]. Mutations occurring at the codons 600 of *BRAF* and 61 of *RAS* genes are common in human thyroid tumors, with these mutations being the most frequent events that lead to the activation of MAPK- and mTOR-signaling pathways. However, they are variably prevalent depending on the histology of the tumor [19,20]. While *BRAF* mutations are exclusive of malignant tumors, most particularly papillary TC (PTC) (50%), *RAS* mutations may occur in both adenomas (20–25% in FAs) and carcinomas (30–45% in follicular TCs (FTCs)). Mutations in *RAS*, are capable of constitutively activating both MAPK- and mTOR-signaling pathways, resulting in the induced activation of the upstream regulators of MAPK (ERK) and mTOR (S6 and AKT) [19,20]. To our knowledge, the role of mutations occurring in *BRAF*, *NRAS*, *HRAS*, and *KRAS* has never been investigated in feline thyroid tumors. Nonetheless, cytoplasmatic RAS expression was reported in cases of feline thyroid hyperplasia and thyroid adenomas [21]. However, in this study a pan-RAS antibody (clone F132) was used, which equally recognizes NRAS, HRAS, and KRAS, making it impossible to determine which specific RAS protein(s) was or were expressed in those tumors [21]. Additionally, as the study did not target phosphorylated (active) RAS proteins, it remains unclear as to whether the detected RAS expression corresponds to functionally active MAPK signaling or merely basal protein presence.

The objective of the present study is to help unravel the genetic dynamics of feline thyroid tumors, with a particular focus on the most commonly activated signaling pathways observed in human TC (MAPK and mTOR pathways), by assessing the immunohistochemical expression of their phosphorylated downstream effectors (ERK, S6, and AKT), and by investigating potential mutations in key driver genes (*BRAF*, *HRAS*, *NRAS*, and *KRAS)*.

## 2. Materials and Methods

### 2.1. Sample Collection

Our series was composed of 15 feline formalin fixed paraffin-embedded (FFPE) thyroid tumor samples diagnosed between 1991 and 2022, retrieved from the archive files of six Portuguese veterinary diagnostic centers. This collection was performed in partnership with the Veterinary Oncology Network (Vet-OncoNet), a Portuguese platform that collects, processes, and centralizes oncological data from participant Portuguese veterinary diagnostic centers [22]. Two additional feline normal thyroid samples were provided by the Histopathological Laboratory of the University of Trás-os-Montes and Alto Douro (UTAD). All of the samples were processed according to standard diagnostic procedures of each center, typically involving fixation in 10% neutral buffered formalin for 24 h.

All of the cases underwent a comprehensive histological revision conducted by a collaborative team of veterinary pathologists (T.B.G. and I.B.) and a human pathologist (S.C.) [1,23]. This reevaluation involved a microscopic examination of 4 µm-thick hematoxylin and eosin slides and adhered to the most up-to-date criteria [1,23], according to which thyroid tumors in cats are considered malignant only in the presence of distant metastasis. Due to restricted access to clinical or follow-up data, definitive classification of the tumors as benign or malignant could not be determined. Therefore, the tumors were classified simply as epithelial thyroid tumors.

FFPE samples were also sectioned for molecular analysis: 10 µm-thick sections were used for DNA extraction and 4 µm-thick sections were used for immunohistochemistry assays. Sample storage and experimental procedures were carried out at Instituto de Investigação e Inovação em Saúde (i3S).

### 2.2. DNA Extraction

Tumoral and normal FFPE tissues underwent DNA extraction using the GRS Genomic DNA Kit BroadRange (GriSP Research Solutions, Porto, Portugal) following the manufacturer’s protocol, which was previously validated in FFPE tissue samples [24,25]. Tissue sections were deparaffinized with xylene (Enzymatic, Santo Antão do Tojal, Portugal) (two incubations of 10 min each) and were hydrated using ethanol (Enzymatic, Santo Antão do Tojal, Portugal) (10 min in 100% ethanol followed by 10 min in 96% ethanol). The tumoral area was then manually micro-dissected into a 1.5 µL Eppendorf tube, with care taken to avoid hemorrhagic and necrotic/autolytic regions previously delineated by the pathologists. Micro-dissected samples were lysed and digested with 200 µL of buffer BR1 and 20 µL of proteinase K (10 mg/mL) (Fisher BioReagents, Proteinase K, Ottawa, ON, Canada). Overnight incubation occurred at 60 °C, under agitation of 750 rpm in the thermoblock. Additional proteinase K and extended incubation were applied as needed. Post-digestion, 200 µL of buffer BR2 was added, and the solution was centrifuged. The samples were then transferred to a clean tube, and 200 µL of ethanol 96% pro-analysis (131085, AppliChem GmbH, Darmstadt, Germany) were added, resulting in the precipitation of the DNA samples. A total of 400 µL of Wash Buffer 1, and 600 µL of Wash Buffer 2 were successively applied and centrifuged, followed by elution buffer. The samples were stored at −20 °C.

### 2.3. Primer Design and Polymerase Chain Reaction (PCR)

To analyze the homologous regions concerning the most commonly mutated genes of human thyroid tumors, a Basic Local Alignment Search Tool (BLAST) search was performed [26]. The human nucleotide sequences of *BRAF* (ENSCAFE00845259256), *NRAS* (ENST00000369535.5), *HRAS* (ENST00000311189.8), and *KRAS* (ENST00000311936.8) used in this analysis were obtained from Ensembl (Ensembl release 107, July 2022), and were investigated in the NCBI feline reference genome (Fca126) [27,28]. Exons 16 of *BRAF* (97%), 2 of *NRAS* (95%), 3 of *HRAS* (89%), and exon 2 (98%) of *KRAS* were identified as the corresponding highly homologous feline exons, and the primers were designed targeting those regions (Table 1). The primers were initially designed using the Primer-Blast tool [29], and validation of the in-silico PCR amplifications was attained using the University of California Santa Cruz In Silico PCR [30]. The primers were fabricated by Integrated DNA Technologies (Belgium).

To amplify all of the regions of interest, the Bioline PCR Kit (MyTaq HS Mix 2X, Memphis, TN, USA) was used during all PCR amplifications, following the manufacturer’s instructions. For each sample, the reaction mixture was composed of 3.50 µL of water, 5.00 µL of MyTaq (Master HS Mix 2X, Memphis, TN, USA), 0.25 µL of each primer pair, and up to 1 µL of DNA at a concentration of 50 ng/µL. The PCR conditions were the same for all primers, consisting of an initiation step of 2 min at 95 °C, followed by 35 cycles, which encompassed denaturation at 95 °C for 30 s, annealing at 55 °C for 30 s, and elongation at 72 °C for 20 s. At the end, a final step at 72 °C for 1 min was employed for a final elongation. The PCR products were visualized under ultraviolet light, using electrophoresis analysis. Then, the PCR products were purified by incorporating 2 µL of the enzyme Exonuclease I (20,000 U, 20 U/µL, Thermo Fisher Scientific, Vilnius, Lithuania) combined with Fast AP Thermosensitive Alkaline Phosphatase (1000 U, 1 U/µL, Thermo Fisher Scientific, Vilnius, Lithuania) and submitting the samples to thermocycling, consisting of one step of 30 min at 37 °C, followed by a 15 min step at 85 °C.

### 2.4. Sanger Sequencing

Sanger sequencing analysis using forward or reverse primers was performed to sequence the human mutation hotspots of *BRAF* (exon 16), *NRAS* (exon 2), *HRAS* (exon 2), and *KRAS* (exon 2), using the kit ABI Prism Big Dye Terminator v3.1 Cycle Sequencing (Fisher Scientific Applied Biosystems, Warrington, UK), and by following the manufacturers’ recommendations. The sequenced fragments were analyzed through capillary electrophoresis using Applied Biosystems sequencers 3130/3130xl Genetic Analyzers (Foster City, CA, USA), and they were visualized in ChromasPro version 2.6.6 (Technelysium-DNA Sequencing Software, Brisbane, Australia). Sequence analysis was conducted through visual inspection in conjunction with the use of MEGAX version 10.2.5 software (Institute of Molecular Evolutionary Genetics, Pittsburgh, PA, USA) [31]. The mutations initially identified were validated in a new independent amplification. The nomenclature of all variants was assigned following the guidelines recommended by the Human Genome Variation Society [32] and was submitted to Mutalyzer for confirmation [33]. The two normal tissues were also analyzed as a control and compared with the tumoral DNA samples.

### 2.5. Immunohistochemical Assays (IHC)

To assess the activation status of MAPK, mTORC1, and mTORC2 immunohistochemical labeling of phosphorylated-ERK Thr202/Tyr204 (pERK), phosphorylated-S6 Ser235/236 (pS6), and phosphorylated-AKT Ser473 (pAKT) was conducted on 16 FFPE samples. The phosphorylated forms of ERK, S6, and AKT were specifically chosen as they have been previously reported as markers of MAPK, mTORC1, and mTORC2 activation [34,35,36]. pERK, pS6, and pAKT were previously validated in other feline tissues [37,38,39]. Since phosphorylation is a key regulatory event for the activation of these pathways, the immunohistochemical detection of these phosphorylated proteins provides a predictive, yet indirect, method to assess their activation status.

The samples, previously deparaffinized in xylene, underwent simultaneous hydration and heat-induced antigen retrieval, using Dewax and HIER Buffer L (TA-999-DHBL, Richard Allan Scientific LLC, Kalamazoo, MI, USA) at 90 °C for 45 min in a steamer, followed by a 20 min cooling period at room temperature (RT). After several PBS washes, the samples were incubated with a Ultravision Hydrogen peroxide block (TA-060-H202Q, Epredia^®^, Kalamazoo, MI, USA) (10 min at RT), followed by PBS washes and incubation with a Ultravision Protein Block (TA-125-PBQ, Epredia^®^, Kalamazoo, MI, USA) (10 min at RT). The primary antibody was incubated in all samples and in the positive control tissues, according to the specified conditions listed in Table 2. The samples were then incubated with the Primary Antibody Amplifier (TL-060-QPB, Epredia^®^, Kalamazoo, MI, USA) for 10 min at RT, and then HRP Polymer Quanto (TL-060-QPH, Epredia^®^, Kalamazoo, MI, USA) was added (10 min). The IHC labeling was revealed by conducting incubation with 3,3-diaminobenzidine (DAB) for 5 min (Table 2). The samples were counterstained with Gill’s hematoxylin, dehydrated, and clarified. Finally, Mounting Medium (4112, Richard-Allan Scientific, San Diego, CA, USA) was employed for sample assembling.

Three observers (A.M., C.T. and T.B.G.) independently evaluated the expression of pERK, pS6, and pAKT using the Axioskop 2 Zeiss microscope (Carl Zeiss, Jena, Germany) and a semi-quantitative method previously described [34]. Each observer assigned scores for two parameters: (1) the extension of immunolabeling, defined as the percentage of stained tumor cells, scored as 0 (<5%), 1 (5–25%), 2 (26–50%), 3 (51–75%), or 4 (76–100%); and (2) the intensity of the staining, scored as 0 (absent), 1 (faint), 2 (moderate), or 3 (strong). For each case, the scores of each parameter were then averaged across the three observers. The final immune reactive score (IRS) was obtained by multiplying the averaged extension and intensity scores, resulting in an immunoreactive score ranging from 0 to 12, which is referred to as immune expression. The expression levels were categorized as negative (IRS < 1), low (IRS 1–3), moderate (IRS 4–8), or high (IRS 9–12). Images were captured using a digital slide scanner (Philips IntelliSite Pathology Scanner 3.2), with the scale bars set at 50 µm and 20 µm for the inset images.

### 2.6. Statistical Analysis

Statistical analyses were performed using GraphPad Prism 9 (Version 9.1.1) and IBM SPSS Statistics (Version 29.0). The available clinical-pathological data (age, sex, tumor size, extrathyroidal extension, multifocality (presence of multiple tumor foci within the same thyroid lobe, hereafter referred to as multifocal tumors), unifocality (presence of a single tumor focus in one thyroid lobe, further referred as unifocal tumor), and vascular invasion, and mitotic count) were examined for associations with molecular features. Significance was set at *p* < 0.050, with borderline significance considered for 0.050 < *p* < 0.100. Graphs were created using GraphPad Prism 9 (Version 9.1.1), and significance levels are represented as * for *p* < 0.05, ** for *p* < 0.01, *** for *p* < 0.001, and **** for *p* < 0.0001.

## 3. Results

### 3.1. Clinical-Pathological Data

The study included 15 epithelial thyroid tumors. Among them, three (*n* = 3/15, 20%) presented histological features of atypia, such as vascular invasion (*n* = 3/4), extrathyroidal extension (*n* = 1/4) and the presence of undifferentiated cells (*n* = 1/4). A separate comparative analysis of this group of cases was therefore proposed, by designating them as tumors with atypia TWA) and the others as tumors with no atypia (TNA) (Table 3).

Most TNA presented a follicular architecture (*n* = 7/11, 73%), resembling the phenotype typically observed in follicular adenomas. The remaining cases exhibited cystic (*n* = 3/11, 27%) or papillary (*n* = 1/11, 9%) patterns. TWA included two tumors exhibiting a follicular pattern (*n* = 2/4, 50%), one (*n* = 1/4, 25%) displaying a compact pattern, and one (*n* = 1/4, 25%) presenting undifferentiated cells. All cases involved European shorthair cats, with no observed sex prevalence for either TNA or TWA. The median age was 11.0 years for cats with TNA and 10.0 years for cats with TWA. TNA had a median size of 3.5 cm, while the size information for TWA was limited to one case, measuring 2.1 cm (Table 3). The clinical-pathological features of the series are listed in Table 3.

### 3.2. Identification of a Novel KRAS Mutation

**Figure 1:**
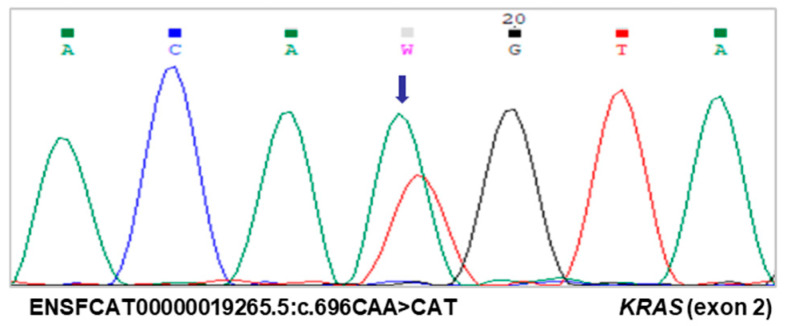
Electropherogram showing the mutation detected in exon 2 of the *KRAS* gene. A single heterozygous mutation in codon 232 (ENSFCAT00000019265.5.696A>T, p.Gln232His) was identified in two tumors of follicular pattern, in a tumor with no atypia, and in a tumor with atypia. Different nucleotides are represented by distinct colored peaks (adenine (A)—green; thymine (T)—red; cytosine (C)—blue; guanine (G)—black). The arrow and letter “W” mark the location of the mutation, with overlapping peaks representing the two alleles of the gene.

The mutational analysis of *KRAS* exon 2 identified a novel *KRAS* mutation in codon 232 (ENSFCAT00000019265.5:c.696A>T (p.Gln232His)) (Figure 1) in two epithelial tumors of the follicular pattern (2/16, 13%), one TNA and one TWA, both presenting multiple tumor foci within the same thyroid lobe. This mutation occurred in a codon homologous to human codon 43 (feline codon 232).

### 3.3. Expression Profiles of pERK, pS6, and pAKT

In most TNA, the activation of the MAPK-signaling pathway was evident, with 10/11 (91%) TNA presenting positive pERK expression (IRS ≥ 1). The distribution of pERK expression in TNA included 9% (*n* = 1/11) with negative, 82% (*n* = 9/11) with low, and 9% (*n* = 1/11) with moderate expression. Additionally, all of the TNA (*n* = 11) showed positive IHC labeling for pS6, with 8/11 (73%) demonstrating moderate expression, 2/11 (18%) low, and 1/11 (9%) displaying high expression. Regarding pAKT, positive expression was identified in 73% (*n* = 8/11) of the TNA. Negative pAKT expression occurred in 27% (*n* = 3/11) of the TNA, low expression in 64% (*n* = 7/11) and moderate in 9% (*n* = 1/11) of the TNA. In TWA, positive expression was noted in pERK, pS6, and pAKT (75%, 75%, and 100%, respectively) (Supplementary Table S1). No expression for any of the proteins was observed in the normal tissues.

**Figure 2:**
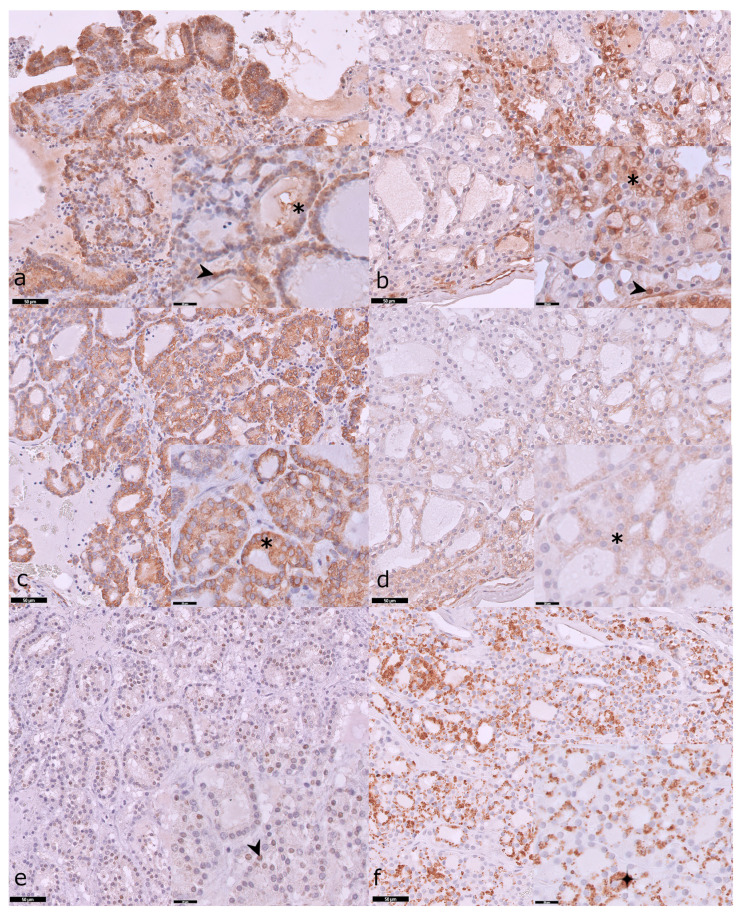
pERK, pS6, and pAKT IHC labeling in tumors with no atypia (TNA) and tumors with atypia (TWA). (**a**) pERK IHC labeling in a TNA of follicular pattern; IHC labeling is primarily cytoplasmic (asterisk) and nuclear (arrowhead). (**b**) pERK IHC labeling in a TWA of compact pattern; IHC labeling is cytoplasmic (asterisk) and nuclear (arrowhead), with expression concentrated at the tumor periphery. (**c**) pS6 IHC labeling in a TNA of follicular pattern, with diffuse cytoplasmic staining (asterisk). (**d**) pS6 IHC in a TWA of compact pattern; diffuse cytoplasmic staining (asterisk), with intensification at the tumor periphery. (**e**) pAKT IHC labeling in a TNA of follicular pattern, with nuclear expression (arrow heads). (**f**) pAKT IHC labeling in a TWA of compact pattern; diffuse cytoplasmic staining (asterisk), with occasional granular cytoplasmic expression (cross).

Among the pERK positive cases in TNA (*n* = 10), expression was predominantly cytoplasmic-nuclear (*n* = 6/10), 60%), followed by cytoplasmic (*n* = 3/10, 30%), and one (*n* = 1/10, 10%) case displayed only nuclear expression (Figure 2a). In TWA, all three pERK-positive cases showed nuclear-cytoplasmic IHC labeling (Figure 2b). pS6 expression was cytoplasmic across all tumors (Figure 2c,d), with cytoplasmic-nuclear immunolabeling observed in two TNA of the follicular pattern. Among the positive cases (*n* = 8), pAKT expression was present in the cytoplasm of most TNA (*n* = 7/8, 88%) (Figure 2e), presenting one TNA with only nuclear expression (*n* = 1/8, 12%) (Figure 2f).

The median values of AKT IRS (expression) were significantly higher in TWA (2.5 ± 4.3), in comparison to TNA (2.0 ± 2.0) (*p* < 0.05). No differences were encountered regarding pERK and pS6 expression in TNA (2.0 ± 0.0, and 6.0 ± 4.0, respectively) and TWA (2.5 ± 4.3, 3.5 ± 10.3, respectively).

### 3.4. Relationship Between the Expression of pERK, pS6, and pAKT and Clinical-Pathological Features of Cats with TNA

**Figure 3:**
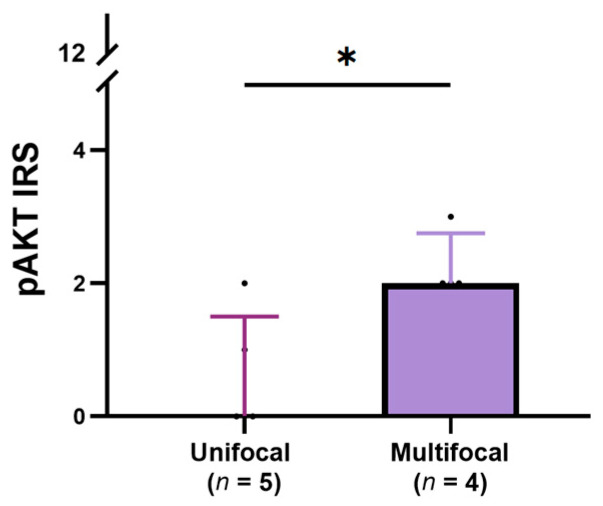
Association between pAKT IRS and the focality of the feline with no atypia (TNA). TNA presenting multiple tumor foci within the same thyroid lobe showed higher pAKT IRS. The results are shown as median ± IQR, and statistical analyses were made using the Mann–Whitney test. **IQR** interquartile range; **IRS** immune reactive score. * *p* < 0.05.

The expression and intensity values were compared with clinical-pathological data to assess the biological role of pERK, pS6, and pAKT in TNA. When the expression of pERK was considered, no significant associations were found, but when using solely intensity, a negative tendency with the tumor’s size was encountered (τ = −0.505, *p* = 0.088). pS6 expression was tendentially negatively correlated with the size of the tumors (τ = −0.546, *p* = 0.0524) (Supplementary Table S2). Greater pAKT expression in TNA was associated with tumors presenting multiple tumor foci within the same thyroid lobe (*p* = 0.027) (Figure 3). No other associations between clinical-pathological data and the expression of pERK, pS6, and pAKT were identified. The presence of the *KRAS* mutation in the TNA of the follicular pattern was associated with higher pS6 intensity (*p* = 0.046) (Table 4). However, its presence was not associated with the expression of pERK (*p* = 0.585), pS6 (*p* = 0.214) nor pAKT (*p* = 0.852).

### 3.5. The Correlation Between pERK, pS6, and pAKT in Tumors with No Atypia

**Figure 4:**
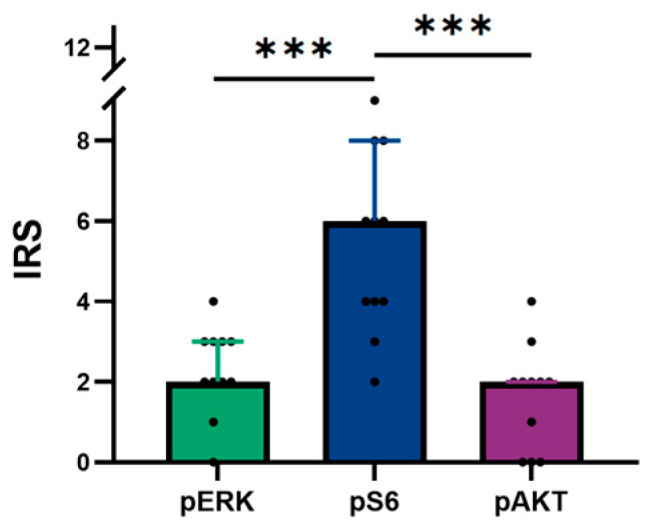
Differential expression pattern of pERK, pS6, and pAKT in feline tumors with no atypia. pS6 IRS was higher than pERK and pAKT. Results are shown as median ± IQR and statistical analyses were made using the Mann–Whitney test. **IQR** interquartile range; **IRS** immune reactive score. *** *p* < 0.001.

No significant correlations were identified in the expression of the three proteins; however, a tendency was observed between pERK and pS6 expression (τ = 0.454, *p* = 0.092). The expression of pAKT and pERK (τ = −0.400, *p* = 0.121) or pS6 (τ = −0.267, *p* = 0.307) did not present any significant correlation. Moreover, pS6 expression was higher than pERK (*p* < 0.001) and pAKT (*p* < 0.001) (Figure 4).

## 4. Discussion

Thyroid carcinoma is rare in both humans and cats. Nevertheless, the occurrence of thyroid nodules is quite common in both species, and thyroid adenomas are the most represented thyroid neoplasia (90–95%) in humans [1,2,3,4,6,40,41]. In the present study, all cases were identified as epithelial thyroid tumors, yet in other reports, adenomas and carcinomas are represented in 95–98% and 2–5%, respectively [1,3,14,41,42,43]. According to actual diagnosis criteria, thyroid tumors in cats are considered carcinomas only in the presence of distant metastasis [1,23], unlike other species such as dogs and humans. In this study we did not have access to clinical or follow-up data, so classification of the cases as adenomas or carcinomas could not be determined. Regardless of this issue, we observed, in four cases, morphological characteristics that in other species are indicative of malignancy, such as vascular invasion, extrathyroidal extension, and the presence of undifferentiated cells [1,23,44,45,46], raising, in our opinion, an interesting reflection about these different classification criteria in cats. Further studies may be needed to fine-tune thyroid diagnostic classification of thyroid tumors in this context.

FAs are the most diagnosed adenomas in the human thyroid gland, while PTCs are the most common malignancy [4,20]. In the present work, we detected a higher prevalence of follicular-pattern tumors among TNA (73% of the TNA), which is consistent with previous reports of feline thyroid tumors [1,43]. Nevertheless, papillary-pattern TWA were not identified in the present study; instead, the follicular (50%) and compact (25%) patterns, and a tumor presenting undifferentiated cells (25%) were the phenotypes identified among the four TWA.

Human thyroid tumors are often characterized by activating point mutations in *BRAF*, *NRAS*, *HRAS*, or *KRAS*, leading to the overactivation of MAPK- and mTOR-signaling pathways [20,34,47,48]. By exploring the feline exon sequences that correspond to the exons that harbor such mutational hotspots in humans, a novel *KRAS* mutation (p.Gln232His) was identified in two tumors of the follicular pattern (one TNA and one TWA). Despite the small sample size in both groups, it may prompt us to question whether this *KRAS* mutation could play a role in a putative follicular transformation, given its presence in both follicular tumors, and due to a theoretical possibility, discussed by some authors, of a malignant transformation occurring in feline adenomas [3,40]. Feline thyroid nodules often manifest as hyper-functional adenomas, autonomously producing thyroid hormones and leading to hyperthyroidism, and it is hypothesized that functional adenomas possibly evolve into malignant forms over time [3,40]. Indeed, some hyperthyroid cats exhibit both adenomatous and malignant lesions within the same thyroid lobe [49]. Furthermore, long-term administration of methimazole, a commonly used drug for managing feline hyperthyroidism, has also been linked to an increased prevalence of TCs in hyperthyroid cats [50]. To better understand the significance of this mutation in the context of feline thyroid disease, larger studies should be conducted to assess its frequency in benign and malignant feline thyroid tumors, and their correlation with hyperthyroidism could also be interesting to investigate. The other investigated genes (*BRAF*, *NRAS*, and *HRAS*) are not likely to be important in the tumorigenesis of the feline thyroid gland, as no mutations were identified in any of the studied cases. However, these players should be investigated in larger series, because our results could be compromised by the small sample size. It is also important to note that, despite only the exon that harbors the human codon 61 being found homologous to a feline *KRAS* sequence (exon 2), exon 1 of the feline *KRAS* should be investigated in future research. The exon 1 of *KRAS* in cats was identified to harbor mutations in 12/13 codons in 2/7 (29%) cases of feline colorectal cancer [51] and 2/3 (67%) cases of feline pancreatic adenocarcinoma [52]. We believe that initiating this comparative investigation by focusing on regions homologous to well-known human mutation hotspots was a relevant first step to assessing potential similarities in tumorigenic mechanisms. However, we acknowledge that restricting the analysis to these limited regions represents a limitation of our study. Future research should explore other regions of these genes to better understand their role in feline thyroid tumorigenesis.

ERK, S6, and AKT are downstream effectors of MAPK-, mTORC1-, and mTORC2-signaling pathways, respectively. By evaluating the phosphorylation levels of these proteins, we addressed the activation status of MAPK- and mTOR-signaling pathways for the first time in feline thyroid tumors.

Although there was a high percentage of positive pERK protein expression levels (91%) in TNA, immune expression was mainly low (82%). In a study of a cohort of human thyroid tumors, pERK expression was detected only in PTCs, while FTCs and FAs did not express pERK [53]. Our study cohort mainly consisted of tumors of the follicular pattern (7 TNA and 2 TWA), so it is possible that in both species ERK activation might not be an important tumorigenic mechanism. Furthermore, higher intensity of pERK was tendentially negatively associated with tumor size in TNA, suggesting that pERK intensity, and maybe MAPK activation, is linked to less aggressive clinical-pathological characteristics in feline tumors.

pS6 expression was positive in all TNA and no associations were observed with clinical-pathological features in feline thyroid tumors. In human PTCs, no association were found between tumor size and pS6 expression; however, it was associated with other less aggressive features, including the absence of tumor capsule invasion, the absence of extrathyroidal extension, clear tumor margins, and the absence of lymphocytic infiltration [35]. Contrarily, the assembly of mTORC2 and, consequently, AKT activation has been recognized as a marker of aggressiveness in human TC [34,53,54]. AKT activation in human PTC, along with its nuclear translocation, has been associated with distant metastasis [34]. Furthermore, in human TC, pAKT expression was detected in 100% of FTCs [53], 42% of ATCs [55], and 51% of PTCs [34]. In the present study, positive pAKT expression was detected in 73% of TNA and 100% of TWA, with TWA presenting higher pAKT expression levels (2.5 ± 4.3), compared to TNA (2.0 ± 2.0). In human follicular tumors, carcinomas tend to present higher rates of pAKT expression compared to adenomas [53]. Additionally, in resemblance to human tumors, the pAKT biological role in thyroid tumors seems to be associated with more atypical clinical-pathological features, as greater pAKT expression was associated with the occurrence of multiple tumor foci within the same thyroid lobe.

Beyond thyroid tumors, the expression and activation of mTOR and MAPK pathways have been investigated in other feline models, though data remain limited. For instance, pAKT expression has been documented in feline mammary carcinomas [37,39], where it associates with aggressive features and shorter survival times [37]. Similarly, both pS6 and pAKT are significantly overexpressed in feline cutaneous squamous cell carcinomas (CSCC), suggesting the mTOR pathway plays a critical role in the pathogenesis of feline CSCC and may represent a potential therapeutic target [56]. In addition, experimental studies show that pERK and pAKT expression increase in feline epithelial cells following papillomavirus infection, which leads to enhanced cellular proliferation and decreased apoptosis [38].

The protein network between MAPK- and mTOR-signaling pathways is very complex and it may include cross-activation or cross-inhibition between each other, depending on each tumor type [16,55,57,58]. In the present study, mTOR activation, through the assembly of mTORC1, was the most activated pathway in TNA, as depicted by the expression levels of pS6, which were significantly higher than pERK and pAKT. Furthermore, pS6 expression tended to be positively correlated with pERK expression, while pAKT expression tended to be negatively correlated with pS6 expression. This evidence suggests that mTORC1 activation could lead to MAPK cross-activation and mTORC2 cross-inhibition in TNA. In human thyroid tumors, mTOR also seem to display a more critical role in thyroid tumorigenesis, as it is the most activated pathway, compared to MAPK [34,53,59,60]. However, mTORC2 activation in human TC is more prominent, and in contrast to our results, it does not affect mTORC1 signaling [34]. Nevertheless, it is important to note that this pattern could be common to feline thyroid carcinomas and TWA, but with only four cases of tumors with atypia (none confirmed as carcinoma), available in this study, such matters remain to be determined. Additionally, the mutation identified on *KRAS* exon 2 was associated with pS6 intensity and possibly mTORC1 activation. Nonetheless, different mechanisms from those activating point mutations in *BRAF* or *RAS* may be triggering such activation. In an in vivo model of human goitrogenesis, hyperactivation of mTORC1 was observed after chronic stimulation by TSH signaling, while AKT remained inactivated, suggesting that the proliferation of human hyperfunctioning thyroid nodules may be enhanced in a TSH-dependent mTORC1 activation [61]. Mutations in *TSHR* have already been identified in the context of feline thyroid disease, more precisely, in the thyroid nodules of hyperthyroid cats [9]; however, the role of *TSH*-related genes have never been explored in feline thyroid tumors.

In summary, despite the limited number of samples, normal tissues, and limited clinical data available, we have shown that feline thyroid tumors, similarly to human thyroid tumors, are characterized by the activation of MAPK- and mTOR-signaling pathways, but lack mutations occurring in the most common hotspots of *BRAF* and *RAS*. Furthermore, MAPK- and mTOR-signaling pathways seem to display similar biological roles to those observed in human thyroid tumors. However, this aspect should be carefully analyzed in future research, as our conclusions were constrained by the number of cases and antibodies available for our analysis. Additionally, the inclusion of two long-archived cases may represent a pre-analytical limitation for immunohistochemistry, although no evident variation on the intensity of the immunolabeling staining was observed.

## 5. Conclusions

The patterns of MAPK and mTOR activation in feline thyroid tumors seem to mirror their human counterparts. mTORC1 stands out as the predominant pathway in feline thyroid tumors, with mTORC2 activation being relatively rare. In feline thyroid tumors, the potential association between pAKT expression and TNA presenting multiple tumor foci within the same thyroid lobe suggests a possible association with more atypical histological features, although further validation is needed. Its prognostic significance, along with that of pERK and pS6, remains unclear, especially given the absence of *BRAF* mutations and the identification of only two *RAS* mutations (both in *KRAS*). However, due to the limited number of cases and the resulting uncertainty of the findings, such potential warrants further validation in future research. Collaborative efforts spanning medical disciplines hold the potential to deepen our insights into thyroid oncobiology, shedding light on the genetic factors unique to each species and each patient.

## Figures and Tables

**Table 1:** Forward and reverse primers used in the PCR reactions ^1^.

Gene_Primer Direction	EN	Sequence (5′–3′)	GL	FS (bp)
*BRAF_Fw*	16	CACCTCAGATATTTTTCTTCACGA	ChrA2:155209670-155209647	151
*BRAF_Rv*		TGGAGAAACAGCCTCAGTTCT	ChrA2:155209520-155209540	
*NRAS_Fw*	2	CCCAGGATTCTTACCGAAAA	ChrC1:97956864-97956845	188
*NRAS_Rv*		GTACCTGTAAAGGTTAATATCTGCAA	ChrC1:97956677-97956702	
*HRAS_Fw*	3	TTATTGATGGCGAGACGTGC	ChrD1:115031688-115031707	147
*HRAS_Rv*		GGATGTCCTCAAAGGACTTGGT	ChrD1:115031834-115031813	
*KRAS_Fw*	2	GGATTCCTACAGGAAACAAGTAGT	ChrB4:58575912-58575889	188
*KRAS_Rv*		AAACCCACCTATAGTGGTGAATATC	ChrB4:58575725-58575749	

^1^ Codon 61 was assessed for all *RAS* genes. The genomic locations are based on the sequences provided by NCBI for the feline (F.catus_Fca126_mat1.0) genome. **EN** exon number; **FS** fragment size; **Fw** Forward; **GL** genomic location; **Rv** Reverse.

**Table 2:** Conditions employed for all antibodies during immunohistochemical procedures ^1^.

Ab	Reference (Company)	BS	CT	Ab Dilution	Ab Incubation	Epitope Revelation (DAB)
**pERK (Thr202/Tyr204)**	4370 (Cell Signaling, Danvers, MA, USA)	Rabbit (Monoclonal)	Feline and canine mammary carcinoma	1:300	Overnight (17 h), 4 °C	TA-125-QHDX, Epedria®, Kalamazoo, MI, USA
**pS6 (Ser235/236)**	4858 (Cell Signaling, Danvers, MA, USA)	Rabbit (Monoclonal)		1:600	Overnight (17 h), 4 °C	K3468, Dako, Santa Clara, CA, USA
**pAKT** **(Ser473)**	66444-Ig (Proteintech, Rosemont, IL, USA)	Mouse (Monoclonal)		1:250	Overnight (17 h), 4 °C	

^1^ All antibodies were diluted in antibody dilutant (TA-125-ADQ, Thermo Scientific Quanto). **Ab** antibody; **BS** biological source; **CT** control tissues; **DAB** 3,3-diaminobenzidine.

**Table 3:** Clinical-pathological features of feline thyroid epithelial tumors.

Clinical-Pathological Features	TNA (*n* = 11/15, 73%)	TWA (*n* = 4/15, 27%)
Age groups	**(*n* = 10)**	**(*n* = 2)**
Median ± IQR	11.0 ± 6.3	10 ± 0.0
Sex	**(*n* = 11)**	**(*n* = 2)**
Female	6 (45.5%)	1 (50.0%)
Male	5 (54.5%)	1 (50.0%)
Tumor size (cm)	**(*n* = 9)**	**(*n* = 1)**
Median ± IQR	3.5 ± 2.2	2.1 ± 0.0
Vascular invasion	**(*n* = 9)**	**(*n* = 4)**
No	9 (100.0%)	2 (50.0%)
Yes	0 (0.0%)	2 (50.0%)
Extrathyroidal extension	**(*n* = 2)**	**(*n* = 2)**
No	2 (100.0%)	1 (50.0%)
Yes	0 (0.0%)	1 (50.0%)
Focality	**(*n* = 9)**	**(*n* = 4)**
Unifocal	5 (55.6%)	2 (50.0%)
Multifocal	4 (44.4%)	2 (50.0%)
Mitosis count per 2.60 mm^2^	**(*n* = 11)**	**(*n* = 4)**
Median ± IQR	1.0 ± 3.0	2.5 ± 7.0

**TWA** tumors with atypia; **TNA** tumors with no atypia; **IQR** interquartile range.

**Table 4:** Association between pS6 immune profile and *KRAS* status in tumors with no atypia.

*KRAS*	pS6 IRS *			pS6 Intensity		
	MR	RS	*p* (U)	MR	RS	*p* (U)
WT (*n* = 9)	5.89	53.0	0.214	5.065	45.5	**0.046**
Mut (*n* = 1)	2.00	2.0	(1.0)	10.50	10.5	(0.5)

* In tumors with IRS < 1, the intensities were considered as 0. The analysis was performed using the Mann–Whitney test. **IRS** immune reactive score; **MR** median rank; **Mut** mutated; **RS** rank sum; **WT** wild-type.

## Data Availability

The original contributions presented in this study are included in the article/Supplementary Material. Further inquiries can be directed to the corresponding author.

## Supplementary Materials

The following supporting information can be downloaded at: https://www.mdpi.com/article/10.3390/vetsci12070617/s1, Table S1: Distribution of the immune reactive scores of pERK, pS6, and pAKT in feline epithelial tumors; Table S2: Correlation between the expression and intensities of pERK, pS6, and pAKT and the clinical-pathological data of tumors with no atypia.

## Abbreviations

The following abbreviations are used in this manuscript:

TWA	Tumors with atypia
TNA	Tumors with no atypia
FA	Follicular (thyroid) adenoma
IRS	Immune reactive score
MAPK	Mitogen-activated protein kinase pathway
mTOR	Mammalian target of rapamycin
pAKT	Phosphorilated v-Akt murine thymoma viral oncogene homolog Ser473
pERK	Phosphorilated extracellular signal-regulated kinase Thr202/Tyr204
pS6	Phosphorilated 40S ribosomal protein S6 Ser235/236
RAS	Rat sarcoma virus protein family
TC	Thyroid carcinoma
