# Supplementary material for: Exploring MAPK and mTOR Pathways in Feline Thyroid Tumors
Source: Vet Sci. 2025 Jun 24;12(7):617. doi: 10.3390/vetsci12070617 (PMC12298209; doi:10.3390/vetsci12070617)
Supplement: Supplementary file 1 [file vetsci-12-00617-s001.zip › vetsci-3662989-supplementary.pdf]

Supplementary files

Exploring MAPK and mTOR Pathways in Feline Thyroid Tumors

Alexandra Monteiro, Tiago Bordeira Gaspar, Inês Borges, Sule Canberk, Mafalda Pinto, Isabel Pires, Paula Soares, Catarina Tavares

Supplementary Table S1. Distribution of the immune reactive scores of pERK, pS6, and pAKT in feline epithelial tumors.

IRS	TNA (*n* = 11)			TWA (*n* = 4)		
	pERK	pS6	pAKT	pERK	pS6	pAKT
0	1 (9.1%)	0 (0.0%)	3 (27.3%)	1 (25.0%)	1 (25.0%)	0 (0.0%)
1	1 (9.1%)	0 (0.0%)	1 (9.1%)	0 (0.0%)	1 (25.0%)	0 (0.0%)
2	4 (36.4%)	1 (9.1%)	5 (45.5%)	1 (25.0%)	0 (0.0%)	1 (25.0%)
3	4 (36.4%)	1 (9.1%)	1 (9.1%)	1 (25.0%)	0 (0.0%)	2 (50.0%)
4	1 (9.1%)	3 (27.3%)	1 (9.1%)	0 (0.0%)	0 (0.0%)	0 (0.0%)
6	0 (0.0%)	3 (27.3%)	0 (0.0%)	1 (25.0%)	1 (25.0%)	1 (25.0%)
8	0 (0.0%)	2 (18.2%)	0 (0.0%)	0 (0.0%)	0 (0.0%)	0 (0.0%)
9	0 (0.0%)	1 (9.1%)	0 (0.0%)	0 (0.0%)	0 (0.0%)	0 (0.0%)
12	0 (0.0%)	0 (0.0%)	0 (0.0%)	0 (0.0%)	1 (25.0%)	0 (0.0%)

The expressions of pERK, pS6, and pAKT was independently evaluated by 3 observers (A.M., C.T., and T.B.G.), who attributed a score from 0 to 4 for the estimated percentage of stained tumor cells and 0 to 3 for the intensity (of the staining). The product of the former two parameters, named immune reactive score (IRS), ranged from 0 to 12 and the results obtained are summarized above. **TNA** tumors with no atypia; **TWA** tumors with atypia; **IRS** immune reactive score.

Supplementary Table S2. Correlation between the expression and intensities of pERK, pS6, and pAKT and the clinical-pathological data of tumors with no atypia.

	**Age**		**Tumor size**		**Mitotic count**	
	**τb**	**p**	**τb**	**p**	**τb**	**p**
pERK IRS	0.407	0.129	-0.330	0,258	0.094	0.728
pERK Intensity	0.027	0.923	-0.505	0.088	-0.519	0.062
pS6 IRS	0.049	0.852	-0.546	0.0524	0.045	0.866
pS6 Intensity	-0.102	0.726	-0.229	0.460	-0.218	0.457
pAKT IRS	-0.229	0.393	0.096	0.738	-0.429	0.114
pAKT Intensity	-0.212	0.435	-0.034	0.907	-0.225	0.413

In tumors with IRS < 1, the intensities were considered as 0. The analysis was performed using the Kendall Tau-b test. **IRS** immune reactive score.
